# The Influence of Low-Energy Impact Loads on the Properties of the Sandwich Composite with a Foam Core

**DOI:** 10.3390/polym14081566

**Published:** 2022-04-12

**Authors:** Andrzej Komorek, Paweł Przybyłek, Robert Szczepaniak, Jan Godzimirski, Marek Rośkowicz, Szymon Imiłowski

**Affiliations:** 1 Department of Aviation, Polish Air Force University, 08-530 Deblin, Poland; p.przybylek@law.mil.pl (P.P.); r.szczepaniak@law.mil.pl (R.S.); s.imilowski4915@wsosp.edu.pl (S.I.); 2 Department of Mechatronics and Aviation, Military University of Technology, 00-908 Warszawa, Poland; jan.godzimirski@wat.edu.pl (J.G.); marek.roskowicz@wat.edu.pl (M.R.)

**Keywords:** sandwich composite, foam core, static strength, dynamic strength, impact properties, interlayer adhesion

## Abstract

Composite materials are widely used in the construction of means of transport. Due to their low density and high stiffness, sandwich composites generate significant interest. The authors conducted static and dynamic tests in order to determine the effect of density and core thickness on the mechanical properties of a sandwich composite. Particular attention was paid to the impact properties of such composites. Herex and Airex polymer foams of different densities were used as cores, whereas the faces were made up of two layers of fabrics: glass and carbon. The matrix base of the tested materials was made of epoxy resin cured with a dedicated hardener. As a result of the study, a significant influence of the core on the strength parameters of the tested spacer materials was found. The examined polymer foams were found to have different adhesive properties, which affected their residual strength after an impact and the nature of destruction of the studied composites. It was observed that sandwich composites with a thicker core of higher density have higher impact strength and resistance to puncture. In the sandwich composites, low-energy impact loads result in damage only to the layer to which the load has been applied and has a core, so repairing such an element is much easier than in classic layered composites without a core. What is very important is that, in contrast to classic laminates, the bottom cover of the composite is not destroyed at low-impact energy values.

## 1. Introduction

Composite materials are widely used in industry, not only in the construction of means of transport, but also modern materials for niche practical applications, such as ablative materials [1], porous materials [2], adhesives [3], building materials [4], etc.

Composite materials are currently widely used in aerospace constructions [5,6]. The most common composites used in the design of aircraft and helicopters are laminates or sandwich composites. For example, certain passenger airplanes have structures consisting of more than 50% different composites [7].

A variation of multilayered composites are sandwich structures [8] built from two types of materials, in which the outer layers are faces (often composites) and the inner layer, or the core, is made of lightweight materials, such as foam, honeycomb structure or others [9,10]. 

The characteristic feature of the sandwich construction is the use of a multilayer skin consisting of one or more high-strength outer layers (faces) and one or more low-density inner layers (core). This definition, which was proposed by Hoffand Mautner in one of the first articles devoted to a sandwich construction, in 1944 [11], is still valid, current and has been taken up in various forms in the works devoted to this type of structure [12]. 

The concept of using sandwich composites to reduce weight was considered by Duleau and Fairbairn as early as in the 1820s [13]; however, it was not until the 1930s and 1940s that sandwich composites were used in real constructions, e.g., an experimental airplane from the interwar period de Havilland Albatross had a hull made of sandwich composites. The development of core materials in structural composites used in aviation started with the use of balsa wood in the 1940s and the use of honeycomb in the 1950s. Along with the development of materials used both for the core and for the facial, a number of works related to both theoretical analysis and experimental research were started to determine the possibility of shaping various properties of sandwich materials through the selection of components. Plantema [14] published a theoretical basis for sandwich structures as early as 1966.

Cores used in load-bearing sandwich structures can be divided into three types of common materials:

- Homogeneous structures—e.g., balsa,
- Network (cellular) structures—e.g., honeycomb structure,
- Heterogeneous structures—e.g., cellular foams.

Especially the development of the latter allowed for the popularization of sandwich composites in various applications. In the case of a foam core, the preparation and shaping of the surface are simple, and the foam surface often adheres well to covers such as fabrics used in polymer laminates, and the joining of the composite elements can be accomplished by bonding. Most foams are available in a wide range of densities and thicknesses, and the first design problem is to choose the core material with the appropriate parameters, because, according to the available results of experimental studies, the properties—especially the mechanical properties—change in some way with the material density. The work [15] discusses the results of tests of sandwich composites differing in the geometrical dimensions of the core and covers (with a core thickness of 6.25, 12.5 and 19 mm and a cover thickness of 0.25, 0.375 and 1 mm, consisting of 2, 3 or 4 layers of carbon fabric in a matrix of cyanate ester resin) and the density of the core materials (aramid honeycomb), but with the same stiffnesses and coefficients of thermal expansion. The results of the experimental tests show that samples with a higher core-to-face layer ratio have a longer microcrack length and a lower tensile strength (flatwise). Therefore, sandwich panels with a thinner facesheet and thicker core are more susceptible to the damage if subjected to the thermal fatigue.)

In Reference [16], the bending strength of sandwich composites was investigated in five different configurations of three layers of PVC foam core and 6 layers of fiberglass fabric reinforcement in an epoxy resin matrix. Moreover, the authors presented the results of high-velocity impact studies with 4 different velocity and impact energies. The results indicate that there is a clear influence of the core density on the mechanical properties of the composite when bending and at high-velocity impact. For three-point and four-point bending, the use of a low-density core in the outer layers reduces the likelihood of damage to the layered structure compared to a uniform core configuration. When considering the use of these types of composites as energy-intensive protection, e.g., to mitigate the effects of an explosion, the use of a (60–80–100) graded core configuration for a sandwich structure with a low-density stepped core foam layer on the impact side brings a significant improvement in protective properties.

Reference [17] presented the results of research on the influence of the aluminum foam core density and the thickness of the composite covers (aluminum foam sandwich (AFS) beam, especially when the aluminum foam core is made in aluminum alloy and the face sheet thickness is less than 1.5 mm beam) on mechanical properties. The presented results showed that the mechanical properties of the tested composite (aluminum foam sandwich (AFS)) are influenced by both the core density and the thickness of the covers—in the case of 0.8 mm–thick covers, the load capacity of the composite increased with increasing density core, while for 1.2 mm–thick covers, the load capacity decreased with increasing core density.

The composites of this type are produced by various methods which can condition their mechanical properties. 

A critical location in sandwich composites is the place where the covers connect to the core [18,19,20]. The connection must be durable enough to withstand the stresses occurring within the two layers. Therefore, the adhesive material and the method of joining the components are crucial for the strength of this type of a composite.

Due to their structure, sandwich materials are significantly lighter than dimensionally identical sandwich composites. They are also characterized by higher stiffness and bending strength, in reference to weight, than other comparable sandwich composites [20,21,22,23,24].

Due to an extremely favorable relationship between weight and strength, spacer materials are very frequently used in construction, sporting goods industry and in the construction of means of transport, especially in floating and flying objects, as a material for the construction of fuselages, beams, wing ribs and stabilizers [25,26,27].

Many components made up of composites can be subjected to low-energy impact loads acting perpendicularly to the surface of the component [28,29]. This type of loading, despite its low energy value, often results in the deterioration of the strength properties of the composite component [7,30]. 

The difficulty of identifying how they deteriorate and fail can be considered a significant issue with spacer materials. The problem in the testing of sandwich composites arises from the fact that damage caused during lateral impact loads often does not develop on the surface, but within the material, due to its specific structure. It can then be assumed that the composite did not suffer any damage, but in fact the core carried the loads caused by the impact and its effects can only be observed inside the material [31,32] in the form of crushing, denting or peeling from the cover. In this case, the defect may remain hidden because, in the sandwich composite, the face often remains intact after an impact. When the core damage remains undetected and the component is under load, the damage may continue to grow, leading even to its total destruction. There are a number of methods for the detection of damage inside sandwich structures [33,34,35,36]; however, a majority of them are laboratory methods that are difficult to use in the process of daily operation, for example, of aircraft.

Due to the damage caused by lateral impact loads, the following distinction can be made:

- BVID—barely visible impact damage,
- VID—visible impact damage.

BVID, as defined by two aircraft manufacturers, Airbus and Boeing, is damage caused by an impact. They are only defined by the depth and area of damage that cannot be detected during a checkup—a visual inspection made in typical lighting conditions at a distance of 1.5 m [37].

A huge number of combinations of materials and architectures are possible today, both for the core and for the skins [38]. However, for aeronautical applications, certification greatly restricts the possibilities. Due to the widespread use of such materials for aircraft skinning, the authors conducted tests on the resistance of sandwich composites to low-energy impact loads to which aircraft composite structures are exposed in everyday operation. This is a crucial feature, due to the fact that, at low energy values (1...3 J), no signs of material destruction can be seen on the side of load application. This is evidenced by the amount of research conducted in the last two decades; for example, in Reference [39], the authors presented historical developments in the study of composite structures under high-velocity impact events, listing 27 works on this subject in various terms. Therefore, such damage is extremely dangerous for composite structures. Due to the fact that composite materials are complex structures and their responses to damage take different forms, the scale of research into the puncture resistance of composites must be much broader than in the case of simple and homogeneous materials used in engineering structures.

So far, many authors have dealt with the issue of research on sandwich composites subjected to low-velocity impact.

Reference [40] presented the results of tests of sandwich composites with a 25.4 mm–thick PVC foam core and carbon fabric covers in an epoxy resin matrix with a thickness of 1.27 mm. The impact of low-velocity impact and the compression after impact (CAI) was investigated, and in Reference [41], foam core sandwich composites in conjunction with various facings composed of glass, stitching glass, carbon, carbon/Kevlar hybrid and Kevlar fabric were fabricated.

In Reference [42], composites with a core thickness of 10 mm, reinforced with a 3.2 mm–thick prepreg material, were subjected to impacts with an energy of 1 to 9 J.

In Reference [43], tests were carried out on a drop hammer by hitting the energies of 7, 17 and 31 J, examining composites with a 13 mm–thick polyurethane foam core reinforced with 8 layers of 1 mm–thick carbon fabric facings. A numerical analysis of the studied cases was also carried out. In these works, the authors examined composites with a thickness greater than 10 mm, and, in addition, the material used for the covers has a thickness significantly smaller than the thickness of the core, without analyzing its possible impact on the tested properties. In the case of low-velocity impact, most authors focus on the analysis of the destruction mechanisms themselves [38,44,45,46,47], additionally examining the residual strength [40,48], and only some authors present a comprehensive assessment of various properties of the tested material [40,49].

In recent years, the intensively developing field of aviation related to UAVs [50] has started to use more and more sandwich materials with a thickness of less than 5 mm, which also results from the analysis of the results of experimental studies by some authors [15,51,52,53,54].

Hence, in the opinion of the authors, there was a need to test this type of materials, additionally analyzing the influence of external layers on the properties of the material.

The research presented in this paper mainly focused on how perpendicularly acting low-energy loads affect the material of sandwich composites with a polyurethane single thin foam core (3–5 mm). It also determined other properties of a sandwich composite with a core of Herex and Airex foams of different densities certified for aerospace applications.

The type of test was selected in such a way as to reflect the operating conditions of the UAV, and the obtained results allowed for the analysis of the suitability of the tested material for its construction, e.g., the Charpy impact test in the edge configuration reflects the case of a UAV collision with terrain obstacles during flight, such as ropes, masts, antennas or also shrubs or trees. On the other hand, the interlayer shear test aimed at determining the adhesion of the core to the facial, allowing for the assessment of the technology of making the sandwich material in terms of the components used. The results obtained in the future will allow for the development of a methodology for the selection of components and optimization of the composition of the composite depending on the intended purpose of the produced material (type of element or even the planned UAV mission).

## 2. Tested Composites

**Figure 1:**
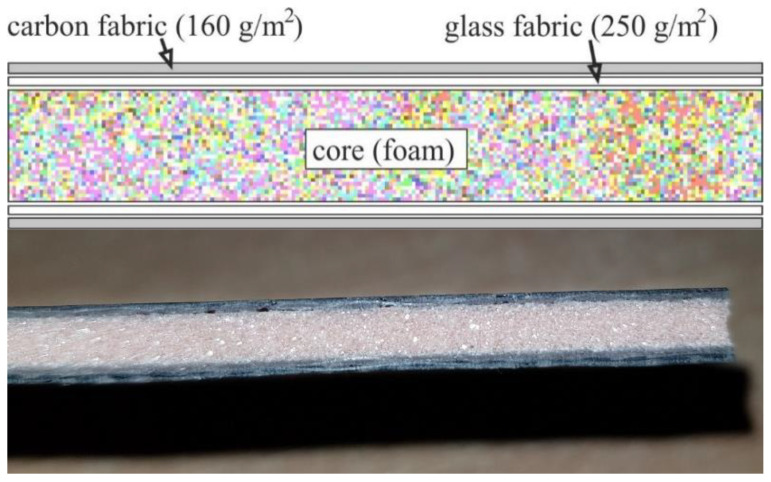
Structure of the sandwich composite used in research.

For the experiments, the authors prepared four sandwich-structured composite boards of Airex and different Herex density by the contact method of manual lamination, using compression on both sides with glass plates, at standard pressure [55]. The facing in each composite consisted of one (external) layer of a carbon fabric 160 g/m^2^ in weight and one (internal) layer of glass fabric 250 g/m^2^ in weight, while the core was Herex foam of the densities 55, 75 and 90 kg/m^3^ (Figure 1) or Airex foam of the 90 kg/m^3^ density. The fabrics in the layers were laid out with fibers parallel to one another.

The matrix base is L 285 MGS epoxy resin certified by the German Federal Aviation Authority, cured with H 285 MGS hardener at the manufacturer’s (Hexion, Stuttgart, Germany) recommended ratio of 100:40. The resin parameters are shown in Table 1.

AIREX C70.90 (Airex AG, Sins, Switzerland ) is a universal structural PVC closed-cell foam with a great rigidity. It is highly durable and has a low water absorption rate, high impact strength and excellent chemical resistance. It can be processed with all commonly used resin systems and processes. Herex (Airex AG, Sins, Switzerland) is a PVC foam made of PVC for the production of sandwich structures. It is suitable for laminating and gluing with epoxy, polyester and vinyl ester. PVC foam shows high product reliability with a relatively low weight in combination with glass, aramid, carbon or other fabrics and resin.

In addition to differences in the density of the composite cores, they also varied in thickness. The Airex foam which was used to make the composite was 3 mm thick, while the Herex foam had corresponding thicknesses: Herex 55—3.8 mm, Herex 75—4.2 mm and Herex 90—5 mm. The resulting thickness of the individual composite panels was as follows: Airex—3.2 mm, Herex 55—4.0 mm, Herex 75—4.4 mm and Herex 90—5.1 mm (Table 2).

In order to assess the mechanical properties of the manufactured composites, it was necessary to prepare samples for an examination. The prepared composite boards were cut down into samples with dimensions depending on the type of the test.

## 3. Experimental Research

Charpy impact tests, puncture-resistance tests and triple support bending tests were carried out as basic tests in order to estimate the residual post impact strength. In order to determine other mechanical properties of the tested materials, standard tensile tests and comparisons of the adhesion forces of the core–face interface for different cores were also carried out.

### 3.1. Impact Strength Testing

**Figure 2:**
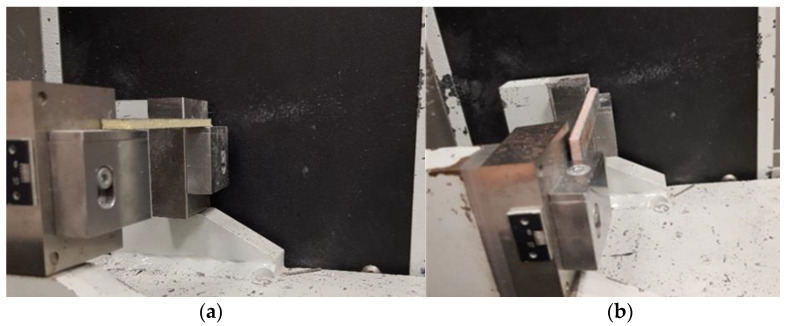
Sample placed for impact testing under: (**a**) edgewise loading and (**b**) flatwise loading.

The impact strength testing of the manufactured sandwich composites was carried out by using two methods—at the edge (1eU) (Figure 2a) and at the surface (1fU) (Figure 2b) of the applied load. The dimensions of the samples used in the study equaled 80 × 10 mm, whereas their thickness was equal to that of the manufactured composites. The samples were un-notched. The investigation was made in accordance with the EN ISO 179-1 standard. The determination of impact strength by means of the Charpy method was performed on the pendulum hammer Galdabini Impact 25 (Galdabini, Cardano al Campo, Italy), with the maximum energy pendulum of 7.5 J. The temperature in the laboratory during the test equaled 20 °C.

**Figure 3:**
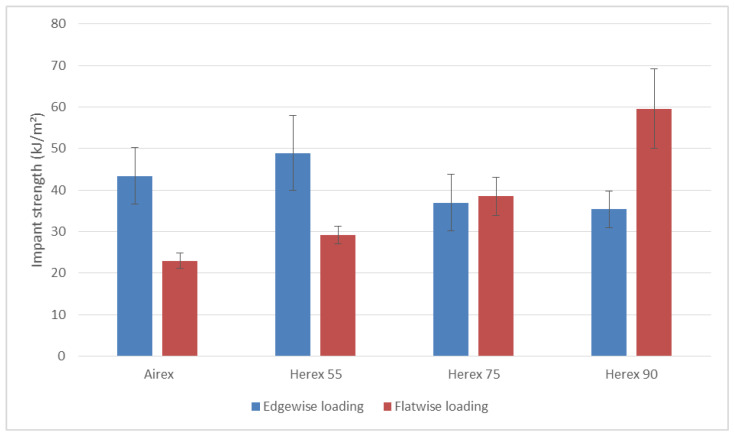
Comparison of impact strength of the tested composites under edgewise and flatwise loading.

Ten samples were tested for each loading configuration and for each composite. The findings of the tests, along with the confidence intervals, are presented in Figure 3.

The composite with the lowest density core (Herex 55) had the highest edgewise-loading impact strength, with an average impact strength of 48.9 kJ/m² ± 9.0 kJ/m² (Figure 3). However, taking into account the confidence intervals, it can be concluded that the impact strengths of the edgewise-loaded samples were comparable. All samples, regardless of the core type, were destroyed in the same way—by incomplete fracture (without a complete separation). 

The highest impact strength at flatwise load application was the Herex 90 core composite (Figure 3)—its value was 59.6 kJ/m² ± 9.6 kJ/m²; the lowest impact strength was the Airex core composite, which was over 65% lower than the impact strength of the Herex 90 core composite. It seems that, at this load, the impact strength is dependent on the thickness of the foam core and less dependent on the density of the foam. For comparison, Herex 90 and Airex C 70.90 foam have the same density, but Airex foam is 2 mm thinner. On the other hand, the difference in impact strength of a composite with a Herex 90 core at a plane impact is over 200% higher. When hitting the edge configuration, the strengths were very similar. It should be noted that, as the thickness of the sandwich element increases, the impact strength of the composite increases in a planar impact configuration, which is much more compatible with the operating conditions and impact loads. In addition, for small foam thicknesses (Airex—3 mm, and Herex 55—3.8 mm), the elements of the facial, i.e., the layered composite, have a much more significant impact on the impact toughness value, as evidenced by its much higher values in the case of the edge impact configuration. Thus, when considering the possibility of application, this type of material should be taken into account already at the design and production stage for the construction of UAV elements exposed to this type of load.

### 3.2. Resistance to Puncture Testing

**Figure 4:**
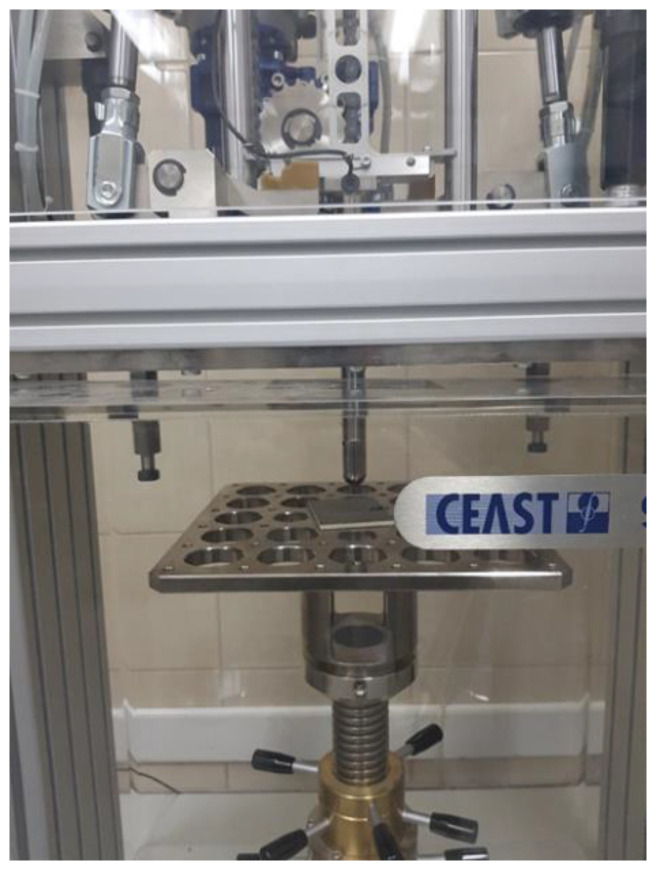
Sample on the table after a conducted test.

The specimens used for examining resistance to puncture had dimensions of 60 × 80 mm and different thickness of the tested composites. The energies with which the samples were tested were as follows: 3, 5, 7, 10 and 15 J. The tests were conducted by using an Instron Chest 9340 drop hammer. The impactor used in the study had a spherical tip with a diameter of 20 mm. The test samples were placed freely on a support with an opening (Figure 4).

**Figure 5:**
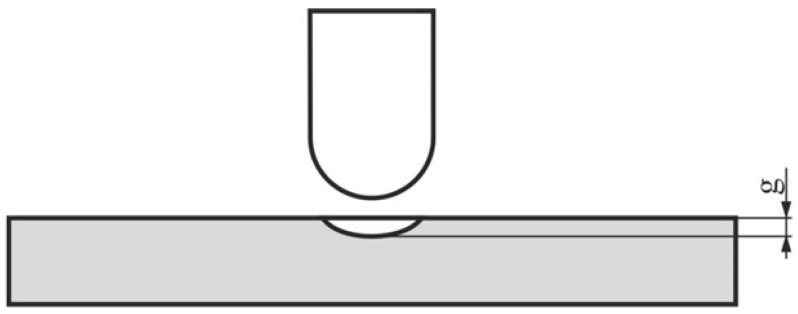
Measured depth of composite damage after concentrated dynamic loading.

Three samples of each type of a composite were tested, using the abovementioned parameters. As damage indicators, the depth of the indentation caused by the impactor loading the sample was measured (Figure 5). The depth was determined by means of a depth micrometer.

**Figure 6:**
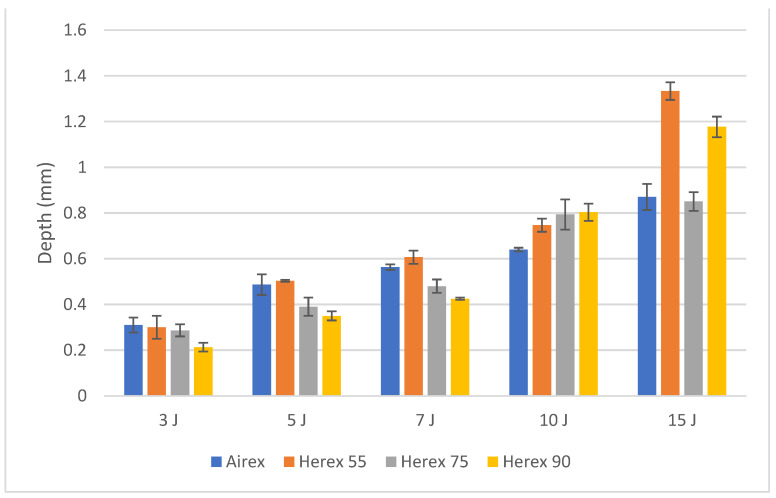
Average indentation depths of all tested composites.

The results of the measured indentation depths are presented in Figure 6.

The value of the damage depth for all foam types increased with an impact energy. At low impact energies of 3 to 7 J, the depth of damage to Herex foam composites was lower for higher-density foams, and this was in line with the expectation that a higher-density foam needs more energy to permanently deform it by the same amount. For higher impact energies, no such dependence was found, and this may indicate a different nature of load absorption, e.g., also by the face on the non-impacted side.

The nature of damage to the investigated sandwich composites was analyzed. The results are listed in Table 3.

**Figure 7:**
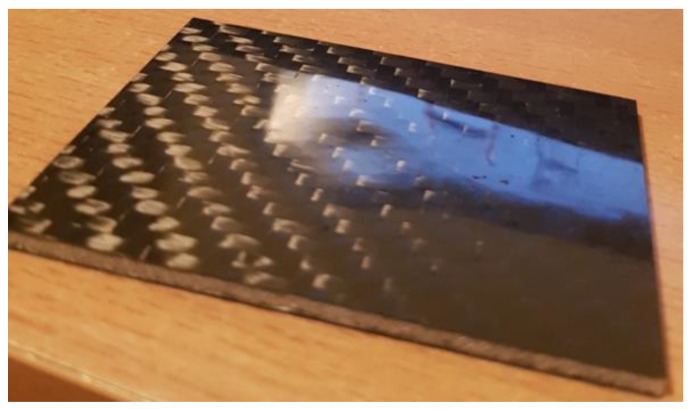
Airex core composite with visible 3 J load mark—from the side of load application.

**Figure 8:**
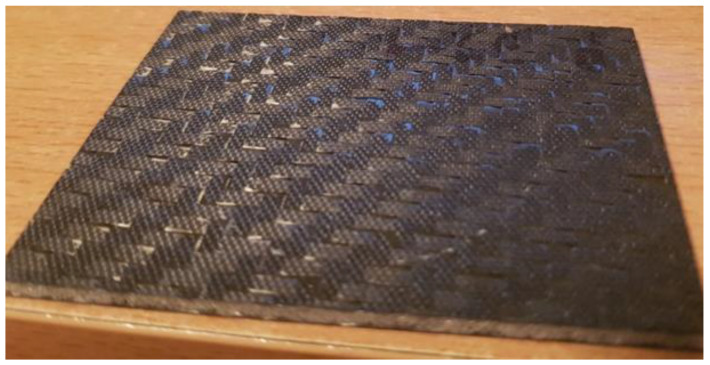
Composite with Airex foam core on the opposite side of the impact—energy 3 J.

As expected, impacts with higher energies caused more damage to the tested sandwich composites. Dents in the impacted face of measurable depth occurred in all cases. For the energy of 3 J, these were only noticeable defects (Figure 7 and Figure 8).

**Figure 9:**
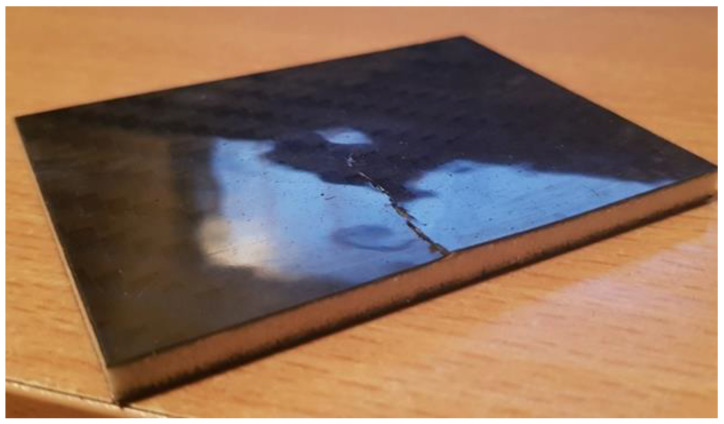
Herex 75 core composite with a crack at the side of energy application equal to 5 J.

In addition to the indentations, cracks appeared in the impacted faces. They increased with higher impact energies (Figure 9).

**Figure 10:**
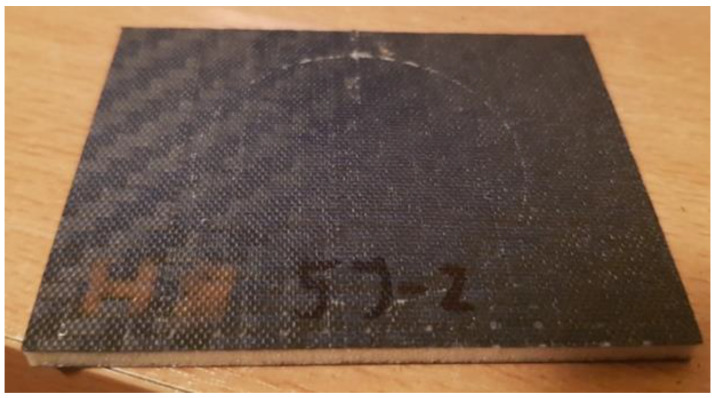
Herex 55 core specimen with face cracking on the side opposite to the impact.

Already at an energy of 5 J, minor damage to the second face was observed in the Herex 55 composite with the smallest thickness (Figure 10).

**Figure 11:**
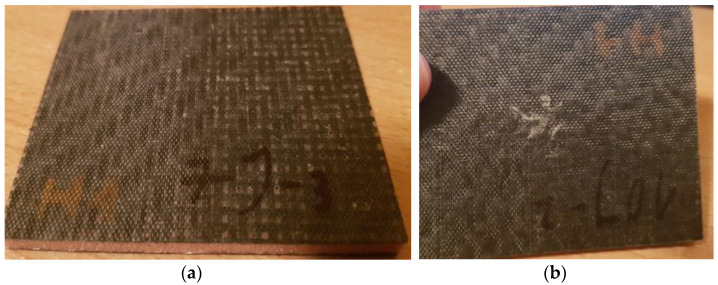
Opposite impact side of composite with Herex 90 core: (**a**) 7 J energy and (**b**) 10 J energy.

For the composite with the Herex 90 core, i.e., the core with the highest thickness and density among the composites tested, only an energy of 10 J caused visible damage to the face surface on the side opposite to the impact (Figure 11a,b). 

At loading energies below 10 J, the Herex 90 core sufficiently dampened the impact. Thus, there was no damage visible to the naked eye on the side opposite to the impacted side. 

**Figure 12:**
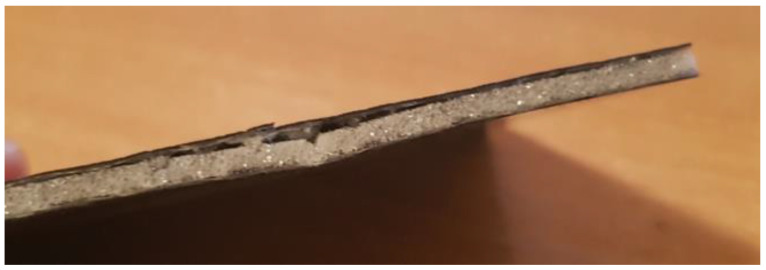
Separated core and face in the composite with Airex core.

In the case of the Airex core composite, an additional type of failure was a separation of the core from the face (Figure 12). This may indicate poorer adhesion properties of the foam.

### 3.3. Bending Strength Testing

The bending test was carried out to determine the Young’s modulus at bending and the bending strength primarily as reference values for determining the residual strength of the tested samples after impact loading. Three-point bending tests were conducted with the traverse movement of 1 mm/min, using a Zwick/Roell 5 kN universal testing machine. The specimens used for this test had dimensions of 60 × 80 mm and a thickness resulting from the thickness of the core. The supports span equaled 64 mm. The sample was centrally loaded with a roller whose diameter equaled 5 mm. The bending strength was calculated from the following formula:

$$\sigma_{f}=\frac{3FL}{2bh^{2}}$$

where *σ_f_* is the bending strength (MPa), *F* is the load (N), *L* is the support span (mm)*, h* is the sample’s thickness (mm) and *b* is the samples’ width (mm)

**Figure 13:**
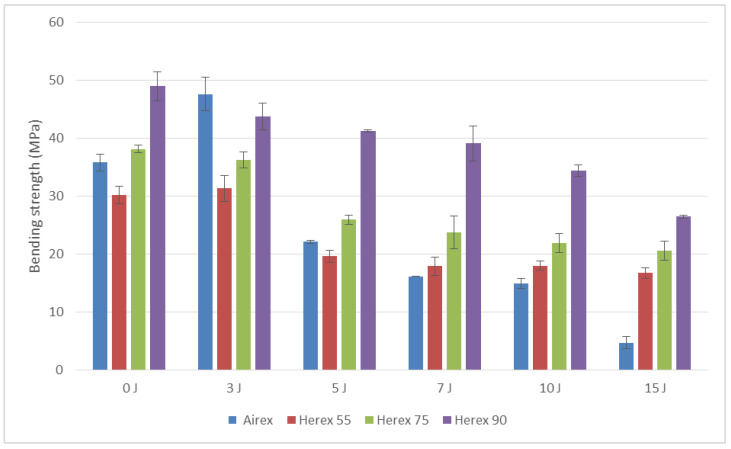
Bending strength of tested composites.

The results of comparative bending strength tests of the samples which were not dynamically loaded and those which were loaded with different energies are shown in Figure 13.

The composite with Herex 90 foam spacer had the highest flexural strength, i.e., Herex 90 foam. When analyzing the test results (Figure 13), it can be concluded that, due to low-energy impact loads, the bending strength of the tested sandwich composites decreases. An impact with an energy of 15 J resulted in a loss of strength of about 50% for the Herex foam composite and up to 90% for the Airex foam composite. (The bending strength after an impact with an energy of 15 J equaled 11% of the strength of the non-impacted composite.)

An impact with an energy 10 J, which can be compared to a release of a tool from the hands of a mechanic operating an aircraft, resulted in a decrease in bending strength of almost 50% for the majority of the examined composites (except for the Herex 90 core composite).

### 3.4. Shear Strength Testing of Cores

**Figure 14:**
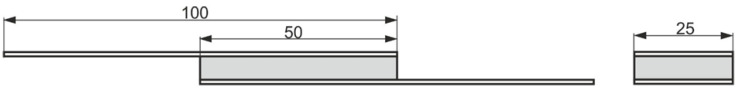
Sample for core strength testing.

**Figure 15:**
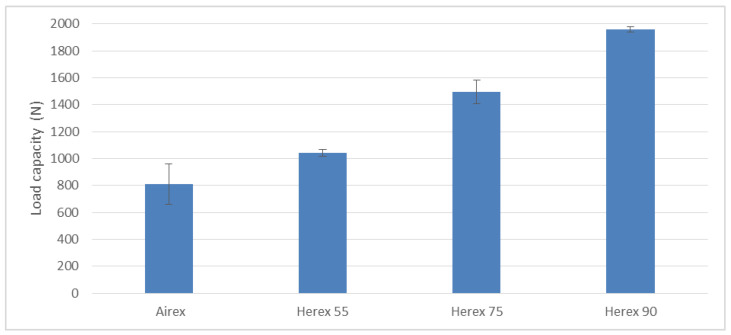
Load capacity of the core or the adhesive joint between the core and the face in the tested composites.

The study was conducted to determine the strength of the core and its adhesion to face. The study was prepared on the basis of modified methodology presented in the paper [56]. The sample drawing is presented in Figure 14. A load pattern analogous to the tensile shear test of glued lap samples was used in the test. It was carried out on a Zwick/Roell 5.0 testing machine with a speed of 2 mm/min. Each series was composed of five pieces. The findings of the tests are shown in Figure 15.

**Figure 16:**
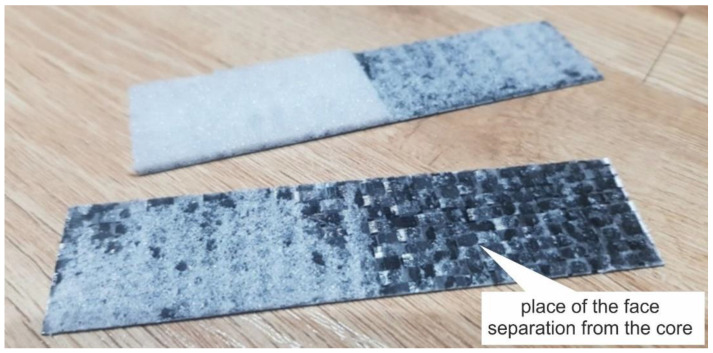
Destroyed sample during an adhesion test between the core and the faces of Airex core composite.

According to the assumptions, the core was expected to separate, which would indicate that the adhesion in the core–cover joint is higher than the core strength. The destruction of the Herex core samples involved cohesive destruction of the foams. The composite with the highest density core Herex 90 showed the highest load capacity (Figure 15). The low strength of the Airex core specimens was due to the adhesive failure of the composite. When this composite was tested, the core was detached from the face (Figure 16).

**Figure 17:**
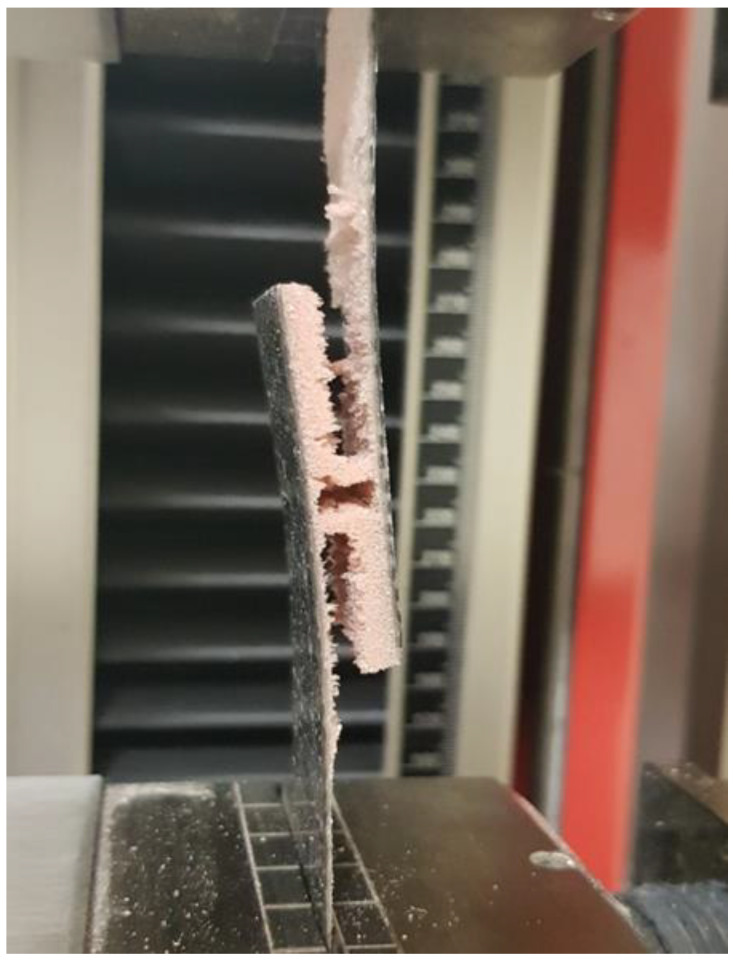
Test sample destroyed in the grip of a Herex core testing machine.

The core itself was not destroyed. There was merely a quick separation of one of the faces. In composites with Herex cores, the core was separated at the edges of the sample, and then the core itself was cohesively destroyed (Figure 17). The tests confirmed the inferior adhesion properties of the Airex foam.

It can be assumed that the greater the density of the core, the denser its structure and the fewer the pores, the greater the adhesion surface is obtained without increasing the size of the samples. Herex 90, as the material with the highest density and the lowest porosity, has an adhesion much better than Herex 75, (and similarly with Airex and Herex 55 cores). Thus, it can be assumed that the greater the porosity of the core, the worse the adhesion on the same surface and with the same resin used and the same covers.

### 3.5. Static Tensile Test

**Figure 18:**
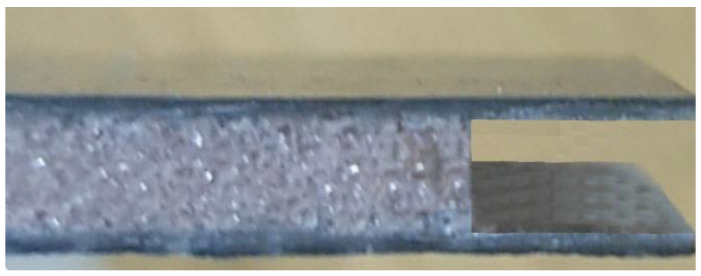
Modified end section of the sample for the tensile strength tests.

For the tests, the blade-shaped samples were modified by removing the end sections of the cores from the specimens on each side and filling the empty spaces (Figure 18) with specially prepared composite inserts.

**Figure 19:**
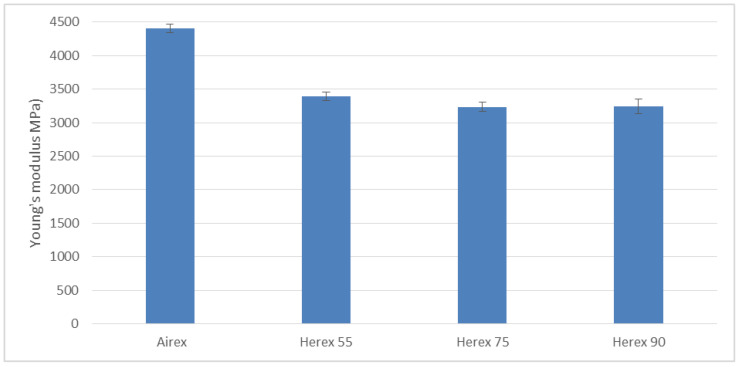
Young’s modulus during tension of the tested composites.

**Figure 20:**
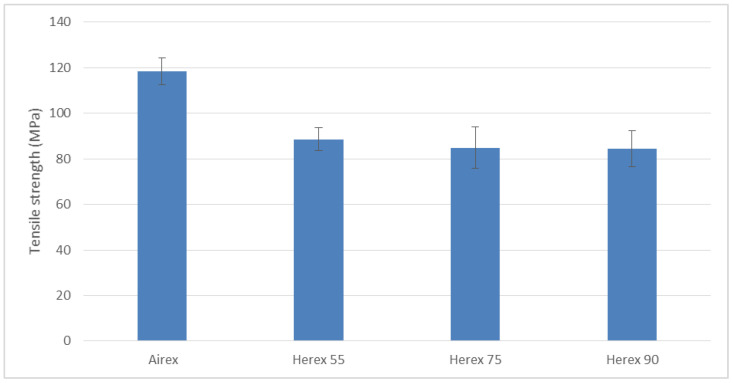
Tensile strength of the tested composites.

Failure to prepare the specimens in this way resulted in their ends being crushed by the jaws of the test machine grips. The solution was successful, as 90% of the samples became destroyed along the measurement length. The results of the findings are presented in the graphs (Figure 19 and Figure 20).

In the graph (Figure 19), it can be observed that the composite with the Airex core has the highest longitudinal modulus of elasticity, and in all composites with Herex cores, its value is at a similar level. 

The obtained results indicate that the composite with Airex foam is a material with greater brittleness than composites with the Herex core, and this may have an influence on the differences in other mechanical properties (e.g., impact strength) of the tested composites.

The strength of the composite with the Airex core was found to be higher, whereas the strength of all materials with Herex cores was found to be very similar (Figure 20). Taking into account the fact that all the examined composites had the same face, it is reasonable to believe that the Herex foam cores do not carry the tensile loads of these composites, while the Airex foam does. The largest value of the module and tensile strength of samples with Ariex filler is due to the relatively large volumetric share of linings in these samples, of about 6.25%.

## 4. Conclusions

Based on the obtained results, the following conclusions were drawn:

(1) As the thickness of the sandwich composite core increases, the impact strength of the composite increases in a plane-impact configuration, which corresponds to impact loads during exploitation. 

(2) Thicker and denser cores provide greater puncture resistance of the composite. It provides better puncture resistance of the composite and better protection against damage to the face on the opposite side of the impact.

(3) Airex foam cores exhibit poorer adhesion properties compared to Herex cores, and this can result in the core being torn off from the face under static and dynamic loads.

(4) The residual strength of the samples after impact loading decreases with the increase of the applied energy (an impact with an energy of 15 J) resulted in a loss of strength of approximately 50% for the Herex foam composite, and in the case of the Airex foam composite, even by approximately 90% (as a result of the separation of the core from the faces). 

(5) The PVC foam core in the sandwich composite absorbs and dissipates the impactor’s energy, which, at low values of impact loads, results in damage only to the layer to which the load was applied and to the core. The bottom layer of the composite remains intact, so repairing such an element is much easier compared to classic layered composites without a core. Materials (with a core) in structures exposed to low-energy impact loads are worth considering. 

(6) Increasing energy resulted in an increase in the damage area of the top layer, and with 3 J energy, visual noticeable deformation of the backsheet of the composite was noticeable. The conducted investigations proved that the density of the PVC core fabric exerts a significant impact on almost all strength properties determined during the tests. It was demonstrated that the density of the spacer fabric affects such parameters as impact strength (1fU method), puncture resistance, bending strength and adhesion between the face and the core.

## Figures and Tables

**Table 1:** L 285 MGS resin parameters.

Property	Value
Density	1.18–1.20 g/cm^3^
Viscosity	600–900 mPas/s
Bending strength	110–120 N/mm^2^
Modulus of rigidity	3.0–3.3 kN/mm^2^
Tensile strength Rm	70–80 N/mm^2^
Compressive strength	120–140 N/mm^2^
Extensibility	5.0–6.5%
Impact strength	45–55^−5^ g/mm^2^
Shore hardness	80–85 D
Curing	24 h at 23 °C
	15 h at 60 °C

**Table 2:** Properties of sandwich composite cores.

Core Type	Designation	Density (kg/m^3^)	Core Thickness (mm)	Composite Thickness (mm)	Percentage by Volume of Face
Airex	Airex C.70.90	90	3.0	3.2	6.25
Herex	Herex 55	55	3.8	4	5
	Herex 75	75	4.2	4.4	4.5
	Herex 90	90	5.0	5.2	2

**Table 3:** Description of damage of sandwich composites.

Core Material and Its Density	Core Thickness (mm)	Extent of Composite Damage				
		Energy 3 J	Energy 5 J	Energy 7 J	Energy 10 J	Energy 15 J
Herex 55 (55 kg/m^3^)	3.8	Indentation in the impacted face. No visible damage to the second face.	Indentation in the impacted face Single cracks at indentation. Small (2–4 mm) cracks in the matrix base of the second face.	Indentation in the impacted face. Single cracks at indentation. Single crack in the second face.	Indentation in the impacted face. Single cracks at indentation. Single crack in the second face.	Denting in the impacted face and radial cracks. Significant losses and slight separation between the core and the face. Single crack in the second face.
Herex 75 (75 g/m^3^)	4.2	Indentation in the impacted face. No visible damage to the second face.	Indentation in the impacted face Single cracks at indentation. No visible damage to the second face.	Indentation in the impacted face. Single cracks at indentation. Single crack in the second face.	Indentation in the impacted face. Single cracks at indentation. Single crack in the second face.	Indentation in the impacted face Single cracks at indentation. Single crack in the second face.
Herex 90 (90 kg/m^3^)	5	Indentation in the impacted face. No visible damage to the second face.	Indentation in the impacted face. No visible damage to the opposite face.	Indentation in the impacted face. Single cracks at indentation. No visible damage to the second face.	Indentation in the impacted face. Single cracks at indentation. Chipping of the matrix and single fiber cracks of the second face.	Indentation in the impacted face Single cracks at indentation. Chipping of the matrix and single fiber cracks of the second face.
Airex (90 kg/m^3^)	3	Indentation in the impacted face. No visible damage to the second face.	Indentation in the impacted face, a single crack at the sample indentation and separation of the core from the face near the edge. Small (2–4 mm) cracks in the matrix base of the second face.	Indentation in the impacted face, a single crack at the specimen indentation and separation of the core from the cover near the edge. Single crack in the second face.	Indentation in the impacted face and a deformation as well as a single crack from the indentation to the edge of the specimen. Significant loss and separation between the core and face near the edge. Single crack in the second face.	Denting in the impacted face, deformation and a single crack to both sample edges, significant losses and separation between the core and the face along the entire length of the edge. Several cracks near the edge of the sample of the second face.

## Data Availability

The raw/processed data required to reproduce these findings cannot be shared at this time, due to technical or time limitations.
